# Entodermoscopy in the Diagnosis of Tinea Nigra: Two Case Reports

**DOI:** 10.5826/dpc.1003a65

**Published:** 2020-06-29

**Authors:** Manuela Lima Dantas, Giovana Serrão Fensterseifer, Paulo Henrique Martins, Irina A. Paipilla Hernandez, Fernando Eibs Cafrune

**Affiliations:** 1 Dermatology, Porto Alegre, Brazil; 2 Dermatology Department, Santa Casa Hospital de Porto Alegre, Brazil

**Keywords:** entodermoscopy, superficial mycosis, tinea nigra, melanocytic lesions

## Introduction

Tinea nigra is a rare superficial phaeohyphomycosis whose characteristic lesion is an asymptomatic, unilateral, well-delimited brown to black macule. Differential diagnoses are melanocytic lesions, subcorneal hemorrhage, and exogenous pigmentation. We present 2 cases of tinea nigra where dermoscopy was helpful in making the correct diagnosis.

## Case Presentations

**Figure 1:**
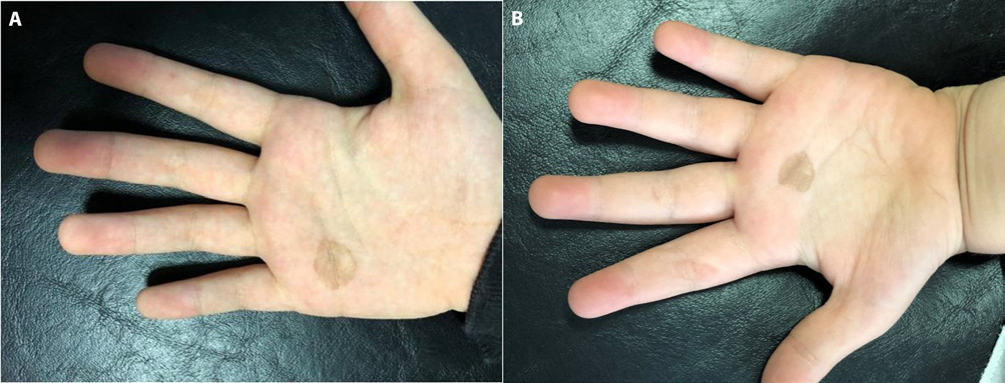
A brown, well-delimited patch on the palm.

**Figure 2:**
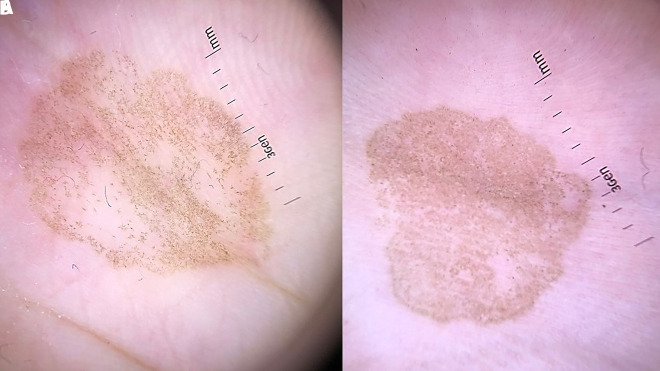
(A) “Brown spicules” forming an almost reticulated patch. (B) “Brown spicules” arranged in the parallel ridge pattern.

An 8-year-old girl presented with a well-defined brown patch on the right palm (Figure 1A). Dermoscopy showed homogeneous “brown spicules” that did not follow the dermatoglyphic lines (Figure 2A).

A 5-year-old boy presented with an irregular patch on the left palm (Figure 1B). Dermoscopy showed “brown spicules” following the parallel ridge pattern (Figure 2B).

**Figure 3:**
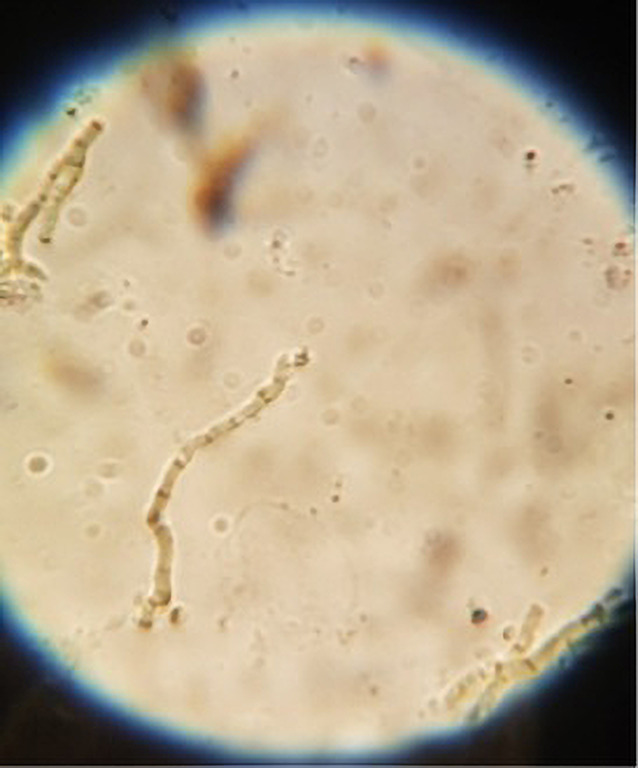
Mycological examination: dematiaceous septate and branched hyphae.

Both patients were from Porto Alegre, Brazil, and had the lesions for approximately 8 months. Mycological examination showed dematiaceous septate hyphae (Figure 3), and *Hortaea werneckii* was identified in culture. Both patients were treated with fenticonazole cream for 30 days and showed clinical improvement.

## Conclusions

Tinea nigra is a superficial fungal infection that affects the stratum corneum. It is characterized by a single unilateral and asymptomatic brown to black patch that affects mainly the palms and, less frequently, the soles of young adults. It is caused by *H. werneckii*, a dematiaceous fungus found in tropical and subtropical climates, mainly in the sand at the beach. Mycological examination shows brown and septate hyphae with thick walls. Examination of the culture shows black, humid, and shiny colonies.

Clinically, tinea nigra can mimic benign and malignant acral melanocytic lesions. However, pigmented melanocytic nevi show a parallel furrow pattern that is not seen in tinea nigra.

The dermoscopy pattern for tinea nigra was first described as “pigmented spicules,” which form an almost reticulated patch [1]. However, Noguchi et al recently described cases of tinea nigra with the parallel ridge pattern, featuring fine, wispy, brown “spicules” (characteristic of tinea nigra) and no color gradation (important clue for differentiation from melanoma) [2]. Besides melanocytic lesions, we have to consider subcorneal hematoma and exogenous pigmentation as differential diagnoses. Dermoscopy of subcorneal hematoma usually reveals reddish black homogeneous areas, often accompanied by satellite globules. In doubtful cases, a scraping test can be performed; gentle scraping off of the stratum corneum with a scalpel will result in partial or complete removal of the pigmentation in cases of subcorneal hematoma. Exogenous pigmentation can present as a parallel ridge pattern on dermoscopy. Previous exposition to some kind of material that can pigment the area, pigmentation favoring foot pressure points, and disappearance within 1 month are important clues for the correct diagnosis (Table 1).

We present 2 cases of tinea nigra, one with the classic dermoscopy of “brown spicules” forming an almost reticulated patch and the other with the most recent dermoscopy pattern described, the parallel ridge pattern, featuring “brown spicules” and no color gradation.

Dermoscopy, a noninvasive technique broadly used in the evaluation of pigmented lesions, has shown to be a useful tool for the diagnosis of tinea nigra.

## Figures and Tables

**Table 1:** Dermoscopy Findings of Tinea Nigra and Its Differential Diagnosis

Tinea nigra	“Pigmented spicules” forming an almost reticulated patch or arranged in parallel ridge pattern with no color gradation
Benign acral melanocytic lesion	Parallel furrow pattern, fibrillar pattern, or lattice-like pattern
Malignant acral melanocytic lesion	Parallel ridge pattern
Subcorneal hematoma	Reddish black homogeneous areas, satellite globules; partial or complete removal of pigment with scrapping test
Exogenous pigmentation	Parallel ridge pattern; correlate with medical history clues
